# A "Barrage" against Disease

**Published:** 1920-12-11

**Authors:** 


### A "Barrage" Against Disease.

Everyone concerned about the health of the community should be interested in the remarkable scheme just outlined by the Ministry of Transport for harnessing the Severn tides by generating electricity over a wide industrial area. It is too obvious to need argument that a well-paid and contented community makes for a higher standard of national health, just as widespread unemployment and industrial unrest open the door to disease and a much lower standard of public health. At the present time we are being invaded by a rapidly rising tide of unemployment, and the cost of production, as well as the uncertainty of employment in almost every branch of industry, are considerably heightened by the growing cost of production of coal and the greater difficulty of "getting" it, as coal, which is, in effect, a diminishing asset, recedes deeper and deeper in the earth and lies in thinner seams. The advantages of the Severn scheme appear to be twofold. It will, in the first place, employ a very large number of men on remunerative production and varied work. It will, in the second place, when completed, substitute water-power for coal, and thus cheapen the initial cost of production in a considerable volume, of industry, and so stabilise employment in the future. It is estimated that the project will cost about £25,000,000, and be the greatest water-power undertaking in the world. If only on the ground of public health, which is the motive and *raison d'être* of this section of The Hospital, we are glad to hear on good authority that the scheme may now be regarded as practical politics. It only remains to receive the definite endorsement of public opinion.

